# Association of C-Reactive Protein with Risk of Developing Type 2 Diabetes Mellitus, and Role of Obesity and Hypertension: A Large Population-Based Korean Cohort Study

**DOI:** 10.1038/s41598-019-40987-8

**Published:** 2019-03-14

**Authors:** Suganya Kanmani, Minji Kwon, Moon-Kyung Shin, Mi Kyung Kim

**Affiliations:** Division of Cancer Epidemiology and Prevention, National Cancer Center, 323, Ilsan-ro, Ilsandong-gu, Goyang-si, Gyeonggi-do 10408 South Korea

## Abstract

This study was undertaken to assess the associations of C-reactive protein (CRP) with incident type-2 diabetes mellitus (T2DM) and to determine the joint effect of obesity and hypertension on them in the large-scale population-based Korean cohort of the Korean Genome and Epidemiology study (KoGES). We included 22,946 men and women from 11 rural communities at baseline (2005–2011). Epidemiological data and blood samples were collected. Incident physician-diagnosed T2DM cases (130 men and 148 women) were self-reported or based on fasting glucose ≥126 mg/dL or HbA1c level ≥6.5% during a median follow-up of 3.0 years (58,916 person-years) between 2007 and 2014. After multivariate adjustment for T2DM risk factors, the hazard ratios for developing T2DM in the highest CRP tertile (T3), compared with the lowest (T1), was 2.80 (1.73–4.52; *p* for trend <0.0001) in women and 1.67 (1.00–2.45; *p* for trend 0.02) in men. The associations between CRP and incident T2DM were more prominent among the older group (≥50 years). And CRP and its combination with obesity and hypertension were associated with increased risk of T2DM. In conclusion, we found positive associations between CRP and incident T2DM in a large population-based Korean cohort.

## Introduction

Type 2 diabetes mellitus (T2DM) is a chronic disease that is considered to be a foremost global health problem, the incidence of which quadrupled within the past 35 years from 108 million in 1980 to 422 million in 2014 and is expected to increase to 552 million by 2030^1^. Korean cohort studies and national surveys likewise have reported increasing prevalence of T2DM in Korea^2,3^. The prevalence of T2DM among Korea adults aged ≥30 years, estimated to be about 8.6 per 100,000 people in 2001, increased to 11.0 per 100,000 people in 2013^4^.

Chronic low-grade inflammation with production of high levels of inflammatory proteins has been implicated in the development of T2DM^5^. C-reactive protein (CRP) is considered to be a prime inflammatory marker of T2DM, which is produced by liver cells, and its expression is regulated by interleukin 6 (IL-6) and TNF-α, which are produced by adipocytes^6^. Chronic inflammation with elevated levels of CRP has been associated with obesity, hypertension, heavy drinking, smoking, and low physical activity^7–14^.

A vast number of cohort studies, having noted elevated levels of CRP in male and female participants^11,12^, suggested that CRP is a risk factor for development of T2DM. The relationship between CRP and T2DM is independent of insulin resistance and body-mass index (BMI)^11,13^. Many studies have elucidated the role of CRP in the development of T2DM, reporting that even after adjustment for BMI, the relationship between CRP level and incidence of T2DM remained statistically significant^11,14–17^. Moreover, observed gender-specific differences have suggested that women have a higher risk of diabetes than men^11,12^. Most studies have been conducted with Western populations, only a relatively small number of studies on Asian populations, with small numbers of participants, having been completed to date. Neither have there been any studies that have investigated the relationship between CRP and incident diabetes in a large population-based Korean cohort. In this context, the aim of the present study was to assess the associations of CRP with risk of incident T2DM and to determine the joint effect of obesity and hypertension in these associations in a large-scale population-based Korean cohort.

## Results

A total of 22,946 participants (men n = 8,537, women n = 14,409) were analyzed in this study. The median concentrations of baseline CRP were higher in men 1.02 mg/L than in women 1.04 mg/L (*P* = 0.0393), respectively. The baseline demographic characteristics, lifestyle factors and metabolic parameters of the study subjects were compared by CRP tertile in men and women, as shown in Table 1. Their lifestyle characteristics and metabolic parameters varied by CRP tertile in both genders. A higher level of CRP was significantly (*P* < 0.0001) associated with lower education, lower income status, higher smoking status, and low physical activity, in both genders. Additionally, a higher level of CRP was significantly (*P* < 0.0001) associated with lower high-density lipoprotein (HDL) cholesterol, lower systolic blood pressure (SBP) and lower diastolic blood pressure (DBP), as well as high BMI, high total cholesterol (TC), high triglycerides (TG) and high glycated hemoglobin (HbA1c) parameters in both men and women. Furthermore, subjects with high levels of CRP showed a higher prevalence of cardiovascular disease (CVD) at baseline.

**Table 1:** Baseline characteristics of study participants by tertiles of C-reactive protein (CRP)^a^.

	Male (n = 8,537)			p^b^	Female (n = 14,409)			p^b^
	Tertiles of CRP (mg/L)				Tertiles of CRP (mg/L)			
	T1 (0.01–0.56)	T2 (0.57–1.34)	T3 (1.35–9.99)		T1 (0–0.43)	T2 (0.44–1.01)	T3 (1.02–9.97)	
CRP, mg/dL	0.35 (0.26–0.46)	0.86 (0.70–1.07)	2.4 (1.76–3.81)	<0.0001	0.27 (0.2–0.35)	0.65 (0.54–0.81)	1.86 (1.32–3.07)	<0.0001
Number of subjects, n	2820	2856	2861		4768	4799	4842	
Age, years	58 (51–65)	59 (51–66)	62 (53–67)	<0.0001	54 (47–63)	59 (51–65)	60 (52–66)	<0.0001
Current smoking, n (%)	794 (32.5)	882 (35)	1024 (40.1)	<0.0001	89 (2.2)	97 (2.3)	124 (2.9)	0.0002
Current drinker, n (%)	1817 (64.6)	1904 (66.8)	1814 (63.5)	<0.0001	1429 (30.1)	1347 (28.1)	1281 (26.6)	<0.0001
Physically active (yes)^c^	895 (31.8)	867 (30.4)	789 (27.7)	0.0021	1485 (31.2)	1450 (30.3)	1375 (28.4)	0.0103
Married, n(%)	2520 (93.3)	2525 (92.5)	2528 (92.5)	0.4659	3732 (81.1)	3590 (77.1)	3469 (74.3)	<0.0001
Education								
<6 years	1159 (41.2)	1256 (44.2)	1380 (48.4)	<0.0001	2772 (58.4)	3215 (67.2)	3512 (72.7)	<0.0001
6–12 years	1256 (44.7)	1223 (43.0)	1221 (42.8)		1660 (34.9)	1379 (28.8)	1170 (24.2)	
≥12 years	396 (14.1)	363 (12.8)	251 (8.8)		319 (6.7)	191 (4.0)	148 (3.1)	
Income (≥3 million won)	276 (15.9)	207 (12.3)	209 (12.2)	<0.0001	434 (15.3)	312 (11.9)	252 (9.4)	<0.0001
BMI, Kg/M^2^								
<18.5	114 (4)	71 (2.5)	85 (3)	<0.0001	203 (4.3)	79 (1.7)	84 (1.7)	<0.0001
1.85–25	1938 (68.7)	1690 (59.2)	1593 (55.7)		3340 (70.1)	2747 (57.2)	2162 (44.7)	
≥25	768 (27.2)	1095 (38.3)	1183 (41.4)		1225 (25.7)	1973 (41.1)	2596 (53.6)	
SBP, mmHg	124 (114–135)	127 (117–139)	129 (118–140)	<0.0001	120 (110–131)	123.5 (111–138)	126 (114–140)	<0.0001
DBP, mmHg	80 (73–88)	81 (75–90)	80 (74–90)	<0.0001	77 (70–84)	79 (70–86)	80 (71–88)	<0.0001
Fasting glucose, mg/dL	93 (88–100)	95 (89–102)	94 (88–102)	<0.0001	90 (85–95)	91 (86–97)	92 (86–99)	<0.0001
Total cholesterol, mg/dL	189 (167–210)	193 (171–216)	194 (170–218)	<0.0001	195 (174–219	203 (180–227)	206 (182–233)	<0.0001
HDL-cholesterol, mg/dL	44 (38–53)	42 (36–50)	41 (35–48)	<0.0001	47 (41–55)	46 (39–53)	43 (38–51)	<0.0001
Triglycerides, mg/dL	118 (85–172)	138 (95–205)	136 (94–203)	<0.0001	102 (76–141)	121 (88–171)	131 (93–186)	<0.0001
HbA1c, %	5.4 (5.1–5.7)	5.4 (5.2–5.7)	5.5 (5.2–5.8)	<0.0001	5.3 (5.1–5.6)	5.4 (5.2–5.7)	5.6 (5.3–5.9)	<0.0001
Incident T2DM, n(%)	33 (1.2)	43 (1.5)	54 (1.9)	0.0871	29 (0.6)	45 (0.9)	74 (1.5)	<0.0001
History of CVD, n(%)	65 (2.3)	77 (2.7)	115 (4.0)	0.0004	74 (1.6)	101 (2.1)	108 (2.2)	0.0397
Family history of diabetes (yes)	229 (8.1)	238 (8.3)	246 (8.6)	0.8083	574 (12)	609 (12.7)	567 (11.7)	0.3253
Family history of hypertension (yes)	475 (16.8)	482 (16.9)	477 (16.7)	0.9757	1061 (22.3)	1008 (21)	1024 (21.2)	0.2663

BMI, body-mass index; SBP, systolic blood pressure; DBP, diastolic blood pressure; HDL, high-density lipoprotein; CRP, C-reactive protein; HbA1c, hemoglobin A1c; T2DM, type 2 diabetes mellitus; CVD, cerebrovascular disease.

^a^Data are presented as number (%), medians (25th, 75th percentiles).

^b^By chi-square test for categorical variable, and Kruskal-Wallis for continuous variables.

^c^Regularity of physical activity was determined according to whether or not subjects participated regularly in any sports to the point of sweating.

We next performed a multivariate Cox regression analysis to corroborate the relationship between CRP level and incident T2DM by gender; the results are summarized in Table 2. During a median follow-up of 3.0 years (58,916 person-years), 278 cases developed T2DM (130 men and 148 women). As seen in Table 2, there were significant associations between CRP and incident T2DM in both men and women. In the multivariable-adjusted model, the HRs for developing T2DM in the highest CRP tertile (T3), compared with the lowest (T1), was 2.80 (1.73–4.52) in women and 1.67 (1.00–2.45) in men, respectively, although the *P*-heterogeneity value (=0.3374) was not significant.

**Table 2:** Multivariate-adjusted hazard ratios (95% CIs) for incident diabetes across tertiles of CRP by gender.

Gender		Tertiles of CRP (mg/L)			p^a^
		T1	T2	T3	
Men	Median (range)	0.35 (0.01–0.56)	0.86 (0.57–1.34)	2.40 (1.35–9.99)	
	Incident case (n)	33	43	54	
	Person-year	7196	7360	7338	
	Model1 [HR (95% CIs)]	1.0 (ref)	1.25 (0.8–1.97)	1.53 (0.99–2.36)	0.0628
	Model2 [HR (95% CIs)]	1.0 (ref)	1.08 (0.67–1.74)	1.67 (1.00–2.45)	0.0272
Women	Median (range)	0.27 (0.01–0.43)	0.65 (0.44–1.01)	1.86 (1.02–9.97)	
	Incident case (n)	29	45	74	
	Person-year	12266	12350	12406	
	Model1 [HR (95% CIs)]	1.0 (ref)	1.45 (0.9–2.31)	2.29 (1.48–3.53)	<0.0001
	Model2 [HR (95% CIs)]	1.0 (ref)	1.58 (0.94–2.65)	2.80 (1.73–4.52)	<0.0001

Model 1: adjusted for age as continuous variable; model 2: additionally adjusted for total cholesterol concentration, resident region, smoking status, alcohol drinking, education, physical activity, family history of diabetes. P is for heterogeneity between men and women using a likelihood test = 0.3374.

^a^p is for the linear trend using median value for each CRP tertile.

**Figure 1:**
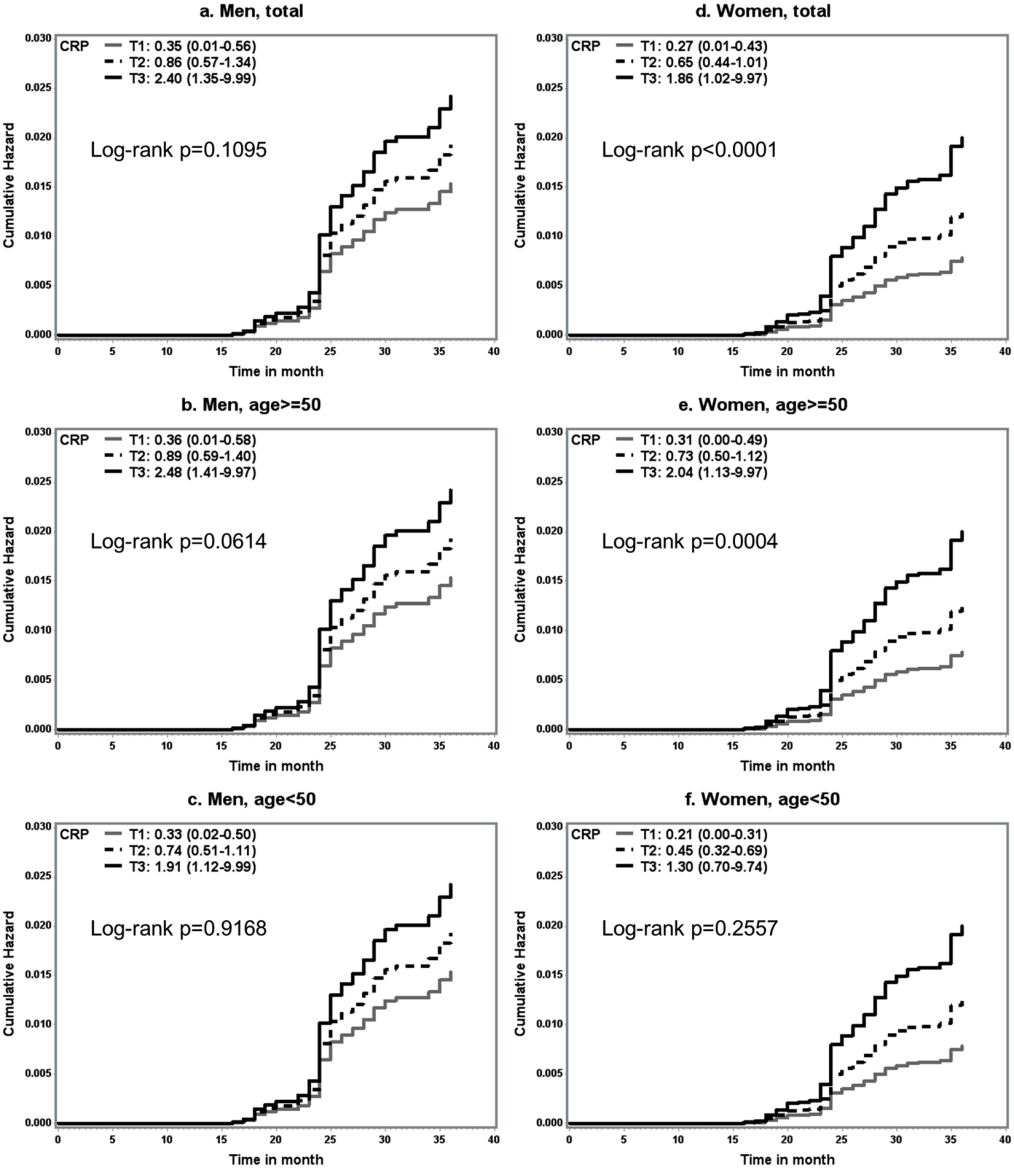
Cumulative hazard plots of type 2 diabetes mellitus (T2DM) by tertiles of CRP (**a**–**c**) men; (**d**–**f**) women). P value was calculated by log-rank test. *Gray solid line* tertile 1; *black dashed line* tertile 2; *black solid line* tertile 3. Legends present tertiles: median (range).

Additionally, we analyzed the relationships between CRP level and incident T2DM by age group (≥50, <50 years) for each gender. As shown in Table 3, the significant associations were more apparent among the older group (aged ≥50 years) than among the younger group (<50 years) for both genders, though the *P*-heterogeneity value between younger and older individuals in each gender was not significant with regard to the association of CRP with incident T2D. The multivariable HR for developing T2DM was 2.95 (1.75–4.98) in the highest CRP tertile (T3) compared with the lowest (T1) among older women, but there was no association with HR across CRP tertiles in younger women. Similarly, the HR for developing T2DM among older men (aged ≥50 years) was 1.83 (1.13–2.97) in the highest CRP tertile (T3) compared with the lowest (T1), but there was no significant association in HR across CRP tertiles in younger men. Figure 1 plots the cumulative hazards for incident T2DM in men (a–c) and in women (d–f) by CRP tertile. There was a progressive increase in the risk of developing T2DM by CRP tertile, especially in older women (log-rank *p* = 0.0004).

**Table 3:** Multivariate-adjusted hazard ratios (95% CIs) for incident diabetes across tertiles of CRP by gender and age.

Gender, age groups	Tertiles of CRP (mg/L)			p^a^
	T1	T2	T3	
Men, ≥50 years				
Median (range)	0.36 (0.01–0.58)	0.89 (0.59–1.40)	2.48 (1.41–9.97)	
Incident case (n)	27	41	49	
Person-year	5874	5943	5985	
Model 1 [HR (95% CIs)]	1.0 (ref)	1.48 (0.91–2.41)	1.76 (1.10–2.81)	0.0332
Model 2 [HR (95% CIs)]	1.0 (ref)	1.31 (0.79–2.19)	1.83 (1.13–2.97)	0.0128
Men, <50 years				
Median (range)	0.33 (0.02–0.50)	0.74 (0.51–1.11)	1.91 (1.12–9.99)	
Incident case (n)	4	5	4	
Person-year	1350	1369	1373	
Model 1 [HR (95% CIs)]	1.0 (ref)	1.24 (0.33–4.61)	0.98 (0.24–3.9)	0.8894
Model 2 [HR (95% CIs)]	1.0 (ref)	1.05 (0.56–4.26)	1.08 (0.26–4.46)	0.9252
Women, ≥50 years				
Median (range)	0.31 (0–0.49)	0.73 (0.50–1.12)	2.04 (1.13–9.97)	
Incident case (n)	25	39	61	
Person-year	9490	9416	9439	
Model 1 [HR (95% CIs)]	1.0 (ref)	1.54 (0.93–2.55)	2.37 (1.49–3.77)	0.0002
Model 2 [HR (95% CIs)]	1.0 (ref)	1.77 (1.01–3.12)	2.95 (1.75–4.98)	<0.0001
Women, <50 years				
Median (range)	0.21 (0–0.31)	0.45 (0.32–0.69)	1.3 (0.70–9.74)	
Incident case (n)	4	10	9	
Person-year	2882	2899	2896	
Model 1 [HR (95% CIs)]	1.0 (ref)	2.31 (0.72–7.37)	2.04 (0.63–6.64)	0.4699
Model 2 [HR (95% CIs)]	1.0 (ref)	2.97 (0.81–10.88)	2.18 (0.57–8.35)	0.6348

Model 1: adjusted for age as continuous variable; model 2: additionally adjusted for total cholesterol concentration, resident region, smoking status, alcohol drinking, education, physical activity, family history of diabetes. P is for heterogeneity between older age (≥50 years) and younger age (<50 years) groups using a likelihood test = 0.3374 (in men) and 0.7699 (in women).

^a^p is for the trend using median value for each CRP tertile.

To assess the joint effect of CRP with BMI and hypertension on the risk of incident T2DM, we applied the multivariate Cox regression hazard model, as shown in Table 4. Both obesity and hypertension interacted with the associations between CRP and incident T2DM. Compared with non-obese men and women with the lowest CRP tertile, the multivariable HRs for risk of incident T2DM among obese men and women with the highest CRP tertile was 2.52 (1.44–4.12) in men and 4.74 (2.43–9.23) in women (*p* for trend <0.0001, *p* for interaction <0.01). Likewise, the multivariable HRs for risk of incident T2DM among hypertensive men and women with the highest CRP tertile, compared with normotensive men and women with the lowest CRP tertile, was 1.76 (0.95–3.28) in men (*p* for trend = 0.0439, *p* for interaction = 0.0572), and 4.58 (2.25–9.15) in women (p for trend <0.0001, p for interaction <0.0001).

**Table 4:** Joint effect of CRP with BMI and hypertension on risk of incident T2DM.

CRP (range)	BMI < 25	BMI ≥ 25	p for trend^b^	p for interaction^c^
**Men**				
T1 (0.01–0.56)	1 (ref)	1.41 (0.68–2.96)	<0.0001	0.0073
T2 (0.57–1.34)	0.83 (0.43–1.6)^a^	1.62 (0.87–3.02)		
T3 (1.35–9.99)	1.01 (0.54–1.89)	2.52 (1.44–4.12)		
**Women**				
T1 (0.01–0.43)	1 (ref)	3.04 (1.33–6.92)	<0.0001	<0.0001
T2 (0.44–1.01)	2.22 (1.07–4.62)	2.40 (1.11–5.15)		
T3 (1.02–9.97)	2.77 (1.32–5.78)	4.74 (2.43–9.23)		
**CRP (range)**	**No hypertension**	**Hypertension**	**p for trend** ^**b**^	**p for interaction** ^**b**^
**Men**				
T1 (0.01–0.56)	1 (ref)	1.47 (0.73–2.98)	0.0439	0.0572
T2 (0.57–1.34)	0.83 (0.41–1.70)	1.47 (0.76–2.81)		
T3 (1.35–9.99)	1.46 (0.77–2.77)	1.76 (0.95–3.28)		
**Women**				
T1 (0.01–0.43)	1 (ref)	2.31 (1.01–5.32)	<0.0001	<0.0001
T2 (0.44–1.01)	1.53 (0.71–3.32)	2.81 (1.32–5.95)		
T3 (1.02–9.97)	2.37 (1.14–4.93)	4.58 (2.25–9.15)		

^a^Adjusted for total cholesterol concentration, resident region, smoking status, alcohol drinking, education, physical activity, family history of diabetes, hypertension (in BMI), BMI (in hypertension); Data are presented as HR (95% CIs).

^b^p is for the trend by assigning an ordinal value to each combination.

^c^p is for the interaction of the multiplicative terms (CRP × BMI, CRP × hypertension).

## Discussion

In this large population-based Korean cohort study, the CRP concentration was associated with increased risk of developing T2DM, and this association was more apparent among the older age group (≥50 years). And CRP and its combination with obesity and hypertension were associated with increased risk of T2DM.

It well known that CRP is a common inflammatory biomarker that is elevated in the blood of subjects with severe inflammation and diseases including T2DM and CVD. A study on a Chinese population reported that the level of CRP was higher in T2DM patients than in normal subjects^18^, and suggested that CRP is an independent predictor of incident T2DM. Some other studies also have shown that higher levels of CRP are positively associated with increased risk of developing diabetes^11,14,15^. However, in the Singapore-based Chinese population study including 571 T2DM cases and 571 matched controls^19^, CRP level was not positively associated with higher risk of incident diabetes. In the EPIC-Norfolk population-based cohort study including 293 diabetes cases and 708 controls^20^, the association between serum CRP level and incident diabetes was not significant after full adjustment for potential confounders including waist-hip ratio, serum adiponection and γ-glutamyltransferase.

In the research and clinical settings, CRP is a more utilizable and reliable marker than other inflammatory markers such as cytokines, due to its relative stability in serum or plasma, its ease of measurement, and the availability of the international standard^13^. The mechanism of the association between CRP and T2DM is still not known in detail. However, there are some explanatory factors including oxidative stress, (which is believed to implicate low-grade inflammation)^21^ and genetic factors such as family history of T2DM^22^. In the present study, we included a large population-based Korean cohort, and the results showed that plasma CRP levels varied by gender, age, socioeconomic and education statuses, lifestyle, and metabolic parameters. After full adjustment for potential confounders, our analysis showed that elevated levels of CRP were associated with increased risk of incident T2DM. Moreover, although higher levels of CRP were found in men (median values of CRP: 0.87 in men and 0.66 in women), the associations between CRP and risk of T2DM were more prominent in women than in men. These results are consistent with other studies showing that elevated CRP level is associated with T2DM in both men and women; however, once again a stronger association was observed with T2DM for women than for men^11,12^. A European study has reported that higher levels of CRP were more strongly and independently associated with increased risk of T2DM in women than in men and that this did not change after stratification by age, smoking or alcohol status, obesity or family history of diabetes^23^. This strong association in women might be due to sex hormones and higher body fat or adiposity percentages in women^12^. In a Mexican study^24^, women with elevated levels of CRP in the highest tertile had an increased risk of T2DM relative to the lowest tertile. After adjustment for BMI and HOMA-IR, the observed T2DM risk was moderately changed^24^. Furthermore, the authors of that study suggested that the relationship between CRP and T2DM risk significantly differs by gender, the women having demonstrated a more significant association than had the men.

We found a significant association between CRP and T2DM risk only among the older group (≥50 years) in both women and men. This significant association was slightly stronger in women than in men, although the *P*-heterogeneity value was not significant. However, there was no significant association in younger men and women, which finding might have been due to the small number of incident-T2DM cases. Similarly, a study on a European population has shown an increased risk of T2DM with higher levels of CRP in women aged 55–74 years old^23^. Another study has reported that the highest-quartile CRP showed a higher risk of incident T2DM compared with the lowest-quartile CRP in middle-aged men^16^.

Indeed, most T2DM patients are relatively obese with higher BMI and insulin-resistance levels^25^. Elevated CRP level is thought to induce insulin resistance through possible mechanisms that include promotion of thrombogenic agent production, activation of complement cascade, enhancement of endothelial adhesion molecules expression, and reduction of endothelial nitric oxide synthase (eNOS)^26–28^. In this context, we examined the relationship between CRP and incident T2DM in relation to obesity and hypertension. We found that both obesity and hypertension interact with the association between CRP and incident T2DM (Table 4). The Jackson Heart Study reported that a higher level of CRP was strongly associated with risk of developing T2DM in non-obese women participants^29^. After a HOMA_IR-_stratified analysis, the association between CRP and T2DM was found to be stronger in non-obese women with HOMA_IR_ < 3.0 (*p* = 0.02 for trend). Furthermore, women with and without hypertension were found to have significantly higher HRs of CRP as compared with men. There was no significant association between CRP and T2DM observed in men who were free of hypertension.

The strength of the present study is that it was a large-scale population-based cohort study for a single ethnic group, that it included a 3-year median follow-up with presumption of lack of recall bias in CRP data, and that it comprehensively measured the potential confounders. However, this study also has some limitations. First, study participants were recruited from 11 rural communities in the National Health Examinee Registry, but only participants willing to consent to participation were enrolled, and more women enrolled than men, which ratio is not representative of the general population. Also, we recruited participants from 11 rural communities at baseline measurement, but the first follow-up survey was conducted for only 6 of 11 communities (15,839 subjects, 56% of the baseline population). These facts might have led to selection bias, which, however, occurs in many prospective cohort studies^30^. Second, notwithstanding the confirmed representativeness of the baseline responder population and the supportive evidence presented by comparisons of health outcomes between KNHANES and national cancer statistics that the KoGES data are generalizable to the Korean population^31^, it is difficult to generalize these results to the general Korean population or other populations. The current epidemiological evidence of causality in the observational study setting remains unclear as to whether CRP is a true causal risk factor for T2DM. Therefore, more carefully designed intervention trials and large-scale prospective studies on different populations are warranted to prove the causality and validate this finding.

In conclusion, we found positive associations between CRP and incident T2DM in a large population-based Korean cohort. These associations were more prominent among the older group (≥50 years), and both obesity and hypertension were found to interact with them. However, further studies on different populations are warranted for corroboration of our findings.

## Material and Methods

### Study Population

The population-based cohorts in the KoGES, including the KoGES_Ansan and Ansung study, the KoGES_Health Examinee (HEXA) study and the KoGES_Cardiovascular Disease Association study (CAVAS), consist of community-dwellers and participants recruited from the National Health Examinee Registry, both men and women, aged ≥40 years at baseline. The KoGES is a large population-based prospective cohort study that is employed to investigate, for long-term follow-up periods, genetic, environmental, and lifestyle determinants of the prevalence and incidence of common complex diseases (i.e. T2DM, hypertension, obesity, metabolic syndrome, osteoporosis, cardiovascular diseases, and cancer) and causes of death among Koreans. The study population consisted of subjects who had participated in the Korean Genome and Epidemiology Study - Cardiovascular Disease Association Study (KoGES_CAVAS). Detailed information on the design and aims of the KoGES can be found elsewhere^31^. In brief, a total of 28,338 community dwellers (10,821 men and 17,517 women aged more than 40 years) were recruited from 11 rural communities in Korea between 2005 and 2011. In eight communities, the study participants were recruited beginning in 2005, and those in the other three communities were recruited beginning in 2006. The first follow-up survey was conducted for 6 of 11 communities, between 2007 and 2014, and the second is ongoing. The present study utilized data from the baseline visit to the first follow-up examination in 2014. Of the original 28,338 participants at baseline, 3,376 who had T2DM (11.9-%), 1463 who had missing CRP data, and 1 who had missing diabetes information were excluded. A further 552 were excluded due to evidence of acute inflammation (CRP ≥10 mg/L). Thus, a total of 22,946 (8,537 men and 14,409 women) were included the final analysis. The total number of observed person-years was 21,894 for men and 37,022 for women, and the number of T2DM incidences was 130 among men and 148 among women.

The study protocol was approved by the institutional review board of Korea NIH, that of each collaborating institution that participated in the KoGES_CAVAS, and that of the National Cancer Center. All of the participants provided written informed consent. All of the methods were performed in accordance with the relevant guidelines and regulations.

### Data collection

All of the study participants underwent an interview and physical examination, and provided biospecimens for the baseline and follow-up examination according to the standard procedures. The participants were questioned by trained interviewers regarding their socio-demographic status, lifestyle (i.e. diet, smoking and drinking statuses, physical activity) reproductive history, psychological stress, social relationships, and disease history (i.e. disease statuses of the participants and his/her family members). For dietary assessment, the semi-quantitative Food Frequency Questionnaire (FFQ) comprising 103 items was developed for the KOGES. Body weight and height were measured with participants wearing light indoor clothing and no shoes. Blood pressure was measured from the right arm, after a rest for at least 5 min in a quiet room, using a standard mercury sphygmomanometer (Baumanometer, W.A. Baum Co. Inc., Copiague, NY, USA). With each subject seated, an appropriately sized cuff, chosen according to mid-arm circumference, was applied snugly around the upper right arm at heart level. Two measurements were taken 5-min apart, and the mean of the two measurements was used for subsequent analyses. Participants who had consumed 400 or more cigarettes were categorized into two groups: former smokers, who abstained from smoking at the time of the questionnaire, and current smokers, who persisted in smoking. Participants were divided according to their alcohol consumption as never drinkers, former drinkers, and current drinkers. Never drinkers were defined as those who had never consumed an alcoholic drink over the course of their lifetime. Former drinkers were defined as participants who abstained from drinking at the time of the questionnaire, while current drinkers were defined as those who persisted in consuming alcohol. Participants were classified in terms of regular exercise based on ‘yes’ and ‘no’ answers to the following question. “Do you currently engage in regular exercise strenuous enough to cause you to break into a sweat at least once per week?” CVD status was collected from the self-reported questionnaire, with participants answering either ‘yes’ or ‘no’ to the question of whether they had been diagnosed with any of the following diseases: coronary artery disease, myocardial infarction, or stroke. Due to a lack of information on menopausal status, women were divided into two groups based on an age cut-off of 50 years.

Blood samples were collected from all of the study participants after at least 10 h of fasting. For long-term storage, both serum and plasma were separated and aliquoted in 6–10 vials (300–500 uL per vial), and all samples were then transported to the National Biobank of Korea^32^ and stored for future research purposes.

Laboratory evaluations were performed in the same core clinical laboratory that is accredited and participates annually in inspections and surveys by the Korean Association of Quality Assurance for Clinical Laboratories. Blood concentrations of glucose, TC, HDL-cholesterol, and TG were measured using the enzyme method (ADVIA 1650 and ADVIA 1800; Siemens Healthineers, Deerfield, IL, USA). Serum concentrations of HDL-cholesterol and TG were determined using enzymatic methods (ADVIA 1650 Chemistry System, Bayer, Leverkusen, Germany). CRP was measured using a turbidimetric assay method (ADVIA 1650 and ADVIA 1800; Siemens Healthineers). HbA1c level was measured by high-performance liquid chromatography (VARIANT II; Bio-Rad Laboratories, Hercules, CA). In addition, the incidence of diabetes was identified by oral glucose tolerance tests, unlike most previous studies.

### Definition of T2DM

The endpoint of this follow-up survey was new-onset T2DM, as determined using WHO-based diagnostic criteria^14^: incident physician-diagnosed T2DM cases were self-reported or based on 8-h fasting plasma glucose (FPG) ≥7.0 mmol/L (126 mg/dL) or HbA1c level ≥6.5% during a median follow-up. Those who had reported currently taking hypoglycemic medications at follow-up were also considered to have new-onset T2DM.

### Statistical analysis

As the basic characteristics based on CRP levels and distributions differed by gender, all of the analysis results were stratified by gender. Blood CRP concentrations were classified into tertile groups of normal subjects (non-diabetes group) for each gender, with cut-off points at 0.56, 1.34 mg/dL for men and 0.44, 1.02 mg/dL for women. Kruskal-Wallis for continuous variables, and Chi-square test for categorical variables were used to compare the characteristics of the study participants at baseline by gender-specific tertiles of CRP. Continuous variables were markedly skewed and therefore were expressed as medians (25th, 75th percentiles). The multivariable Cox proportional hazard model was applied to assess the association of baseline CRP with new-onset T2DM. The HRs were calculated as 95% confidence intervals, and the two-sided probability values were <0.05 statistically significant. Model 1 was adjusted for age; model 2 was additionally adjusted for total cholesterol, smoking habits and alcohol consumption, education, family history of diabetes, and region. In addition, the HRs were calculated by age group (≥50 years, <50 years). We assessed heterogeneity between subgroups (men and women, older age (≥50 years) and younger age (<50 years)) using a likelihood ratio test comparing Cox proportional hazard models with and without interaction terms for CRP and gender or age (≥50 years, <50 years)^33^. The cumulative hazard plot was visualized by gender and age group, and a log-rank test was used to make comparisons between the CRP tertiles. To determine the combined effect of CRP and BMI obesity status or hypertension status, an interaction term was evaluated by including it in the Cox hazard model. P values for the linear trend and for interaction also were calculated. To confirm the assumption of proportional risk, all of the models were evaluated and deemed to be consistent with a model that included time-dependent covariates. All of the statistical analyses were performed with SAS 9.4 (SAS Institute, Cary, NC, USA). P values < 0.05 were considered statistically significant.
